# Effect of Rare Earth Ions on the Properties of Composites Composed of Ethylene Vinyl Acetate Copolymer and Layered Double Hydroxides

**DOI:** 10.1371/journal.pone.0037781

**Published:** 2012-06-05

**Authors:** Lili Wang, Bin Li, Xiaohong Zhao, Chunxia Chen, Jingjing Cao

**Affiliations:** Heilongjiang Key Laboratory of Molecular Design and Preparation of Flame Retarded Materials, College of Science, Northeast Forestry University, Harbin, People's Republic of China; University of Minho, Portugal

## Abstract

**Background:**

The study on the rare earth (RE)-doped layered double hydroxides (LDHs) has received considerable attention due to their potential applications in catalysts. However, the use of RE-doped LDHs as polymer halogen-free flame retardants was seldom investigated. Furthermore, the effect of rare earth elements on the hydrophobicity of LDHs materials and the compatibility of LDHs/polymer composite has seldom been reported.

**Methodology/Principal Findings:**

The stearate sodium surface modified Ni-containing LDHs and RE-doped Ni-containing LDHs were rapidly synthesized by a coprecipitation method coupled with the microwave hydrothermal treatment. The influences of trace amounts of rare earth ions La, Ce and Nd on the amount of water molecules, the crystallinity, the morphology, the hydrophobicity of modified Ni-containing LDHs and the adsorption of modifier in the surface of LDHs were investigated by TGA, XRD, TEM, contact angle and IR, respectively. Moreover, the effects of the rare earth ions on the interfacial compatibility, the flame retardancy and the mechanical properties of ethylene vinyl acetate copolymer (EVA)/LDHs composites were also explored in detail.

**Conclusions/Significance:**

S-Ni_0.1_MgAl-La displayed more uniform dispersion and better interfacial compatibility in EVA matrix compared with other LDHs. Furthermore, the S-Ni_0.1_MgAl-La/EVA composite showed the best fire retardancy and mechanical properties in all composites.

## Introduction

Layered double hydroxides (LDHs) are a kind of layered materials that consists of positively charged layers and the interlayer exchangeable anions [1], [2]. Their general formula can be represented as 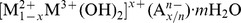. In recent years, LDHs have received considerable attention because of their application as catalysts [3], [4], ion exchangers [5], adsorbents [6], [7], corrosion resistance films [8], precursors [4], [9], drugs [10], [11] and LDHs-polymer composites [12]–[15]. In particular, the use of LDHs as polymer halogen-free flame retardants is a rather promising field of application [16]–[18].

Ethylene vinyl acetate (EVA) copolymer with different vinyl acetate (VA) contents are widely used in the wire, cable, wrapper, adhesive and drug industry [19]–[22]. However, EVA resins are particularly flammable, and its subsequent combustion gives off large volumes of toxic smoke. A feasible solution to this problem is the addition of flame retardant which may improve the fire safety of EVA. Therefore, as a promising non-halogenated additive, LDHs have been applied in EVA for having flame retardancy [23]–[26].

It is well known that good interfacial compatibility between inorganic LDHs and polymer is a very important factor for the improvement of properties of LDHs/Polymer composite. To the best of our knowledge, to improve the dispersion and compatibility of inorganic LDHs phase with polymer matrix, most studies concentrate on the organo-modified treatment of LDHs so far [15], [16], [27]. The reason is because the organo-modifiers make the organic-inorganic hybrid LDHs more hydrophobic. However, although organo-modified treatment can enhance the hydrophobicity of LDHs, the use of large amounts of organic modifiers is not only detrimental to the flame retardancy and the mechanical properties of LDHs/polymer composites due to the existence of large amounts of carbon, but also being not environment-friendly. Therefore, the addition of a small amount of organic modifier combined with other effective way to improve the hydrophobicity of LDHs are feasible.

Rare earth (RE) has been the object of considerable scientific and technological interest due to their distinctive optical [28]–[30], electronic [31], [32], magnetic [33], [34], anticorrosive [35], [36] and catalytic properties [37]. Many of them, especially La and Ce, were used in LDHs materials to enhance the catalytic efficiency [38]–[40]. However, the effects of rare earth elements on the hydrophobicity of LDHs materials and the compatibility of LDHs/polymer composite have been relatively seldom reported.

In our previous report, Ni-containing MgAl-LDHs showed obviously higher flame retardant efficiency in comparison with alone MgAl-LDHs in the EVA matrix [41]. Therefore, for comparison purpose, a small amounts of stearate sodium surface modified Ni-containing LDHs and RE (La, Ce, Nd)-doped Ni-containing LDHs were synthesized by a coprecipitation method coupled with the microwave hydrothermal treatment in the present paper. The main aim of this study focuses on the effects of doped trace amounts of rare earth ions on the amount of water molecules, the crystallinity, the morphology, the hydrophobicity of RE-doped modified Ni-containing LDHs and the adsorption of modifier in the surface of LDHs. Moreover, the effects of interfacial compatibility between LDHs and EVA on the flame retardancy and the mechanical properties of EVA/LDHs composites also were investigated in detail.

## Results and Discussion

### Thermal Analysis of LDHs

**Figure 1:**
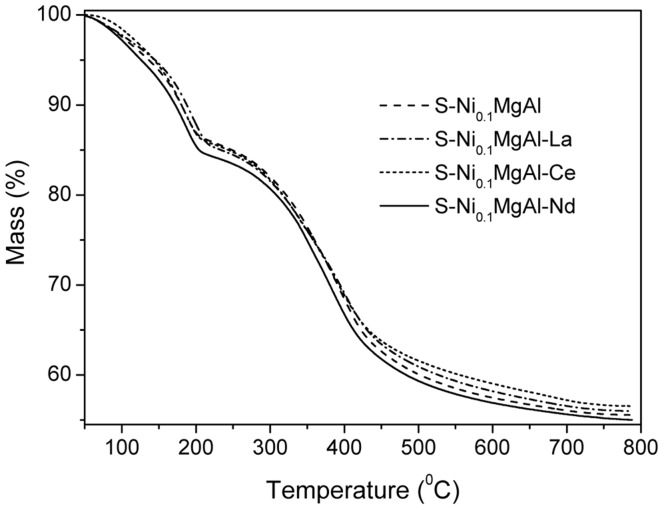
TGA curves of S-Ni_0.1_MgAl, S-Ni_0.1_MgAl-La, S-Ni_0.1_MgAl-Ce and S-Ni_0.1_MgAl-Nd.

Figure 1 illustrates the TGA curves of S-Ni_0.1_MgAl and RE-doped modified LDHs S-Ni_0.1_MgAl-La, S-Ni_0.1_MgAl-Ce and S-Ni_0.1_MgAl-Nd, respectively. As shown in Figure 1, on heating, all LDHs mainly underwent two stages decomposition. The first stage corresponds to the loss of physical absorbed water and interlayer water in the range of 50 to 230°C [42]. The mass losses of water molecules for S-Ni_0.1_MgAl, S-Ni_0.1_MgAl-La, S-Ni_0.1_MgAl-Ce and S-Ni_0.1_MgAl-Nd are 14.2%, 15.0%, 14.5% and 16.0%, respectively. This result indicates that S-Ni_0.1_MgAl-Nd has the most water molecules in all of the above LDHs. The second stage in the range of 230 to 800°C is associated with the dehydroxylation of the metal hydroxide layers, the degradation of carbonates and stearate in LDHs [43]. This stage relates to the mass losses which are 30.3%, 29.0%, 28.9% and 29.0% for S-Ni_0.1_MgAl, S-Ni_0.1_MgAl-La, S-Ni_0.1_MgAl-Ce and S-Ni_0.1_MgAl-Nd, respectively. The above thermal analysis data point out that the addition of the rare earth ions causes different contents of water molecules and stearate in LDHs.

### Powder XRD Characterization of LDHs

**Figure 2:**
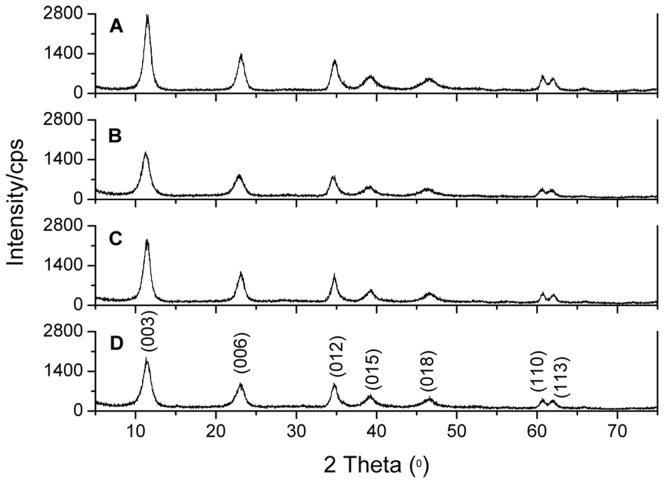
XRD patterns of the synthesized LDHs. A: S-Ni_0.1_MgAl, B: S-Ni_0.1_MgAl-Nd, C: S-Ni_0.1_MgAl-Ce and D: S-Ni_0.1_MgAl-La.

Figures 2 A-D show the XRD patterns of S-Ni_0.1_MgAl, S-Ni_0.1_MgAl-La, S-Ni_0.1_MgAl-Ce and S-Ni_0.1_MgAl-Nd, respectively. All patterns exhibit (003), (006), (012), (015), (018), (110) and (113) characteristic reflections of the LDHs structure, which can be indexed in a 3R polytype [44]. These reflection positions are in good agreement with those of the reported unmodified LDHs [44], [45]. This result indicates that the stearate was uptaken on the surface of LDHs rather than intercalated into the interlayer. The basal spacings (*d*_basal_) of S-Ni_0.1_MgAl-La, S-Ni_0.1_MgAl-Ce, S-Ni_0.1_MgAl-Nd and S-Ni_0.1_MgAl, which are calculated from the (003) reflections, are 7.74, 7.71, 7.86 and 7.65 Å, respectively. As expected, these values also confirm that the interlayer of all LDHs are 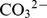 besides 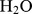 and 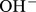, without the coexistence of 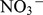 or 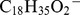 [46]. Furthermore, the doublet corresponding to planes (110) and (113) (recorded in the 2θ range of 60–63°) becomes distinguishable, suggesting a well ordering within the brucite-type layers [47]. However, by carefully observing, it can be found that the addition of rare earth ions reduces the reflection intensity of LDHs (see Figure 2), especially for S-Ni_0.1_MgAl-Nd in comparison with S-Ni_0.1_MgAl. This result indicates that S-Ni_0.1_MgAl-Nd has the lowest crystallinity in all of the above LDHs [48].

From the values of 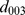, 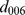 and 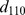 of the reflections in the XRD patterns, the lattice parameters *a* and *c* can be calculated. Furthermore, the 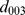, 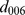, 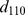, the lattice parameters *a* and *c* of all LDHs are listed in Table 1. The parameter *a* depends on the chemical composition of the metal cations in the layers. It corresponds to the average closest metal-metal distance within a layer, and is calculated as twice the position of the plane (1 1 0). As seen from Table 1, the *a* values of S-Ni_0.1_MgAl-La, S-Ni_0.1_MgAl-Ce and S-Ni_0.1_MgAl-Nd are larger than that of S-Ni_0.1_MgAl due to the addition of rare earth ions. This result confirms that the rare earth cations La, Ce and Nd were introduced to the brucite-type layers. The possible reason is that the radii of rare earth ions are much larger than that of Al^3+^. It leads to a tiny deformation of the structure of RE-doped Ni-containing LDHs compared with S-Ni_0.1_MgAl. The lattice parameter *c*, which is the thickness of crystal cell, can be calculated based on the following equation [49].

**Table 1:** XRD data and structural parameters of all LDHs samples.

Sample	*d*_003_ (nm)	*d*_006_ (nm)	*d*_110_ (nm)	*a* (nm)	*c* (nm)	*L*_003_ (nm)	*L*_110_ (nm)
S-Ni_0.1_MgAl	0.765	0.385	0.1520	0.3040	2.30	8.23	22.35
S-Ni_0.1_MgAl-La	0.774	0.386	0.1526	0.3052	2.32	7.41	26.73
S-Ni_0.1_MgAl-Ce	0.771	0.385	0.1525	0.3050	2.31	9.22	26.80
S-Ni_0.1_MgAl-Nd	0.786	0.387	0.1524	0.3048	2.34	8.27	22.28

The *c* values of S-Ni_0.1_MgAl-La, S-Ni_0.1_MgAl-Ce and S-Ni_0.1_MgAl-Nd are slightly larger than that of S-Ni_0.1_MgAl. Namely, the interlayer region of S-Ni_0.1_MgAl is smaller than those of the RE-doped modified LDHs. An important reason is due to the effect of the amount of water molecules in the interlayer of LDHs, as described by Wypych et al. [50]. As expected, S-Ni_0.1_MgAl has the least water. On the contrary, the RE-doped LDHs have more water. Especially, S-Ni_0.1_MgAl-Nd has the most water based on the TGA analysis.

The crystallite sizes of all of the above LDHs can be calculated by using Debye-Scherrer formula [51].

(2)Where *L* is the crystallite size of the LDHs, *λ* is the wavelength of the radiation used, *B*(*θ*) is the full width at half maximum and *θ* is the Bragg diffraction angle. Table 1 lists the results of the crystallite sizes of all LDHs *L*_003_ in the direction *c* and *L*_110_ in the direction *a*. These results indicate that all of the above LDHs show the nanometer crystallite size.

### FT-IR Spectroscopy of LDHs

**Figure 3:**
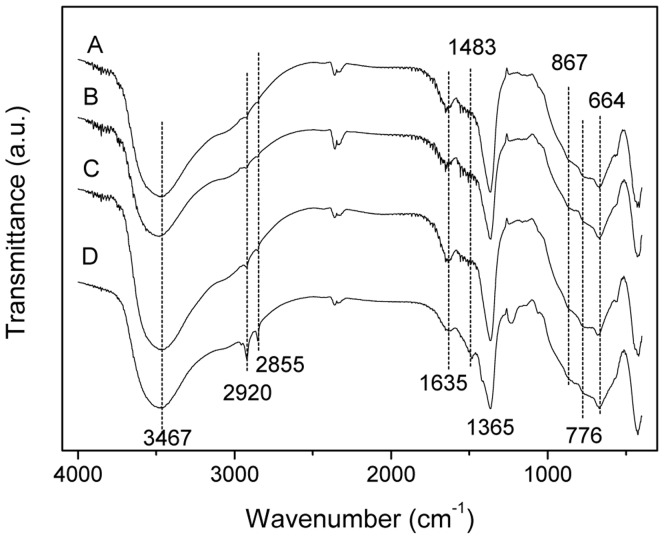
IR spectra of the synthesized LDHs. A: S-Ni_0.1_MgAl, B: S-Ni_0.1_MgAl-Nd, C: S-Ni_0.1_MgAl-Ce and D: S-Ni_0.1_MgAl-La.

Figures 3 A-D illustrate the FT-IR spectra of S-Ni_0.1_MgAl, S-Ni_0.1_MgAl-Nd, S-Ni_0.1_MgAl-Ce and S-Ni_0.1_MgAl-La, respectively. All LDHs show a broad and intense band between 4000 cm^−1^ and 3000 cm^−1^ due to the OH stretching vibration of layer hydroxyl groups and interlayer water molecules. The bands at near 1635 cm^−1^ are originated by the bending mode of interlayer water molecules. The sharp and intense bands at 1365 cm^−1^ are due to the antisymmetric stretching mode of interlayer carbonate [52]. Furthermore, the weak bands at 1483 cm^−1^ are assigned to the bridged-bidentate complexation of interlayer in the above four LDHs [53], [54]. At the same time, the two bands around 867 cm^−1^ and 664 cm^−1^ are characteristic for the *ν*_2_ (out-of-plane deformation) and the *ν*_4_ (in-plane bending) of interlayer carbonate [55]. In the low wave number region between 400 cm^−1^ and 1000 cm^−1^, a series of bands at 776, 562 and 422 cm^−1^ are ascribed to condensed groups, the translation and deformation of M-OH in the brucite-like layers [56]. In addition, all LDHs also display two weak bands at 2920 cm^−1^ and 2855 cm^−1^ which are associated with the C-H stretching vibrations of carboxylate group. Therefore, IR results confirm that a little stearate was uptaken on the surface of LDHs. Moreover, it should be noted that the intensity of C-H stretching bands for S-Ni_0.1_MgAl-Ce and S-Ni_0.1_MgAl-La are the stronger than those of S-Ni_0.1_MgAl and S-Ni_0.1_MgAl-Nd, especially for S-Ni_0.1_MgAl-La. This observation indicates that S-Ni_0.1_MgAl-La has the better adsorption for stearate at the same reaction condition due to the addition La ions.

**Figure 4:**
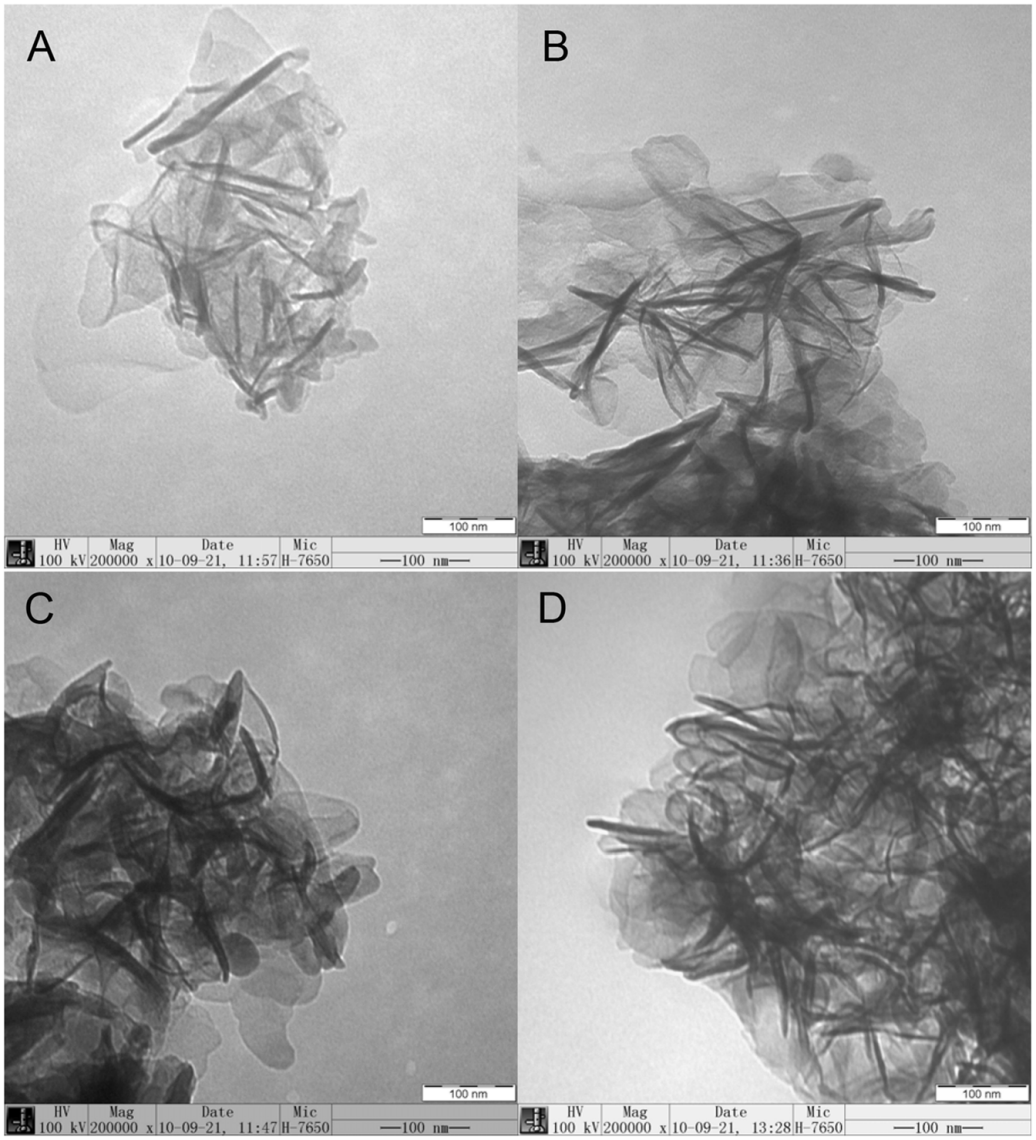
TEM images of the synthesized LDHs. A: S-Ni_0.1_MgAl, B: S-Ni_0.1_MgAl-Nd, C: S-Ni_0.1_MgAl-Ce and D: S-Ni_0.1_MgAl-La.

### Morphology and Contact Angles Analysis of LDHs

**Figure 5:**
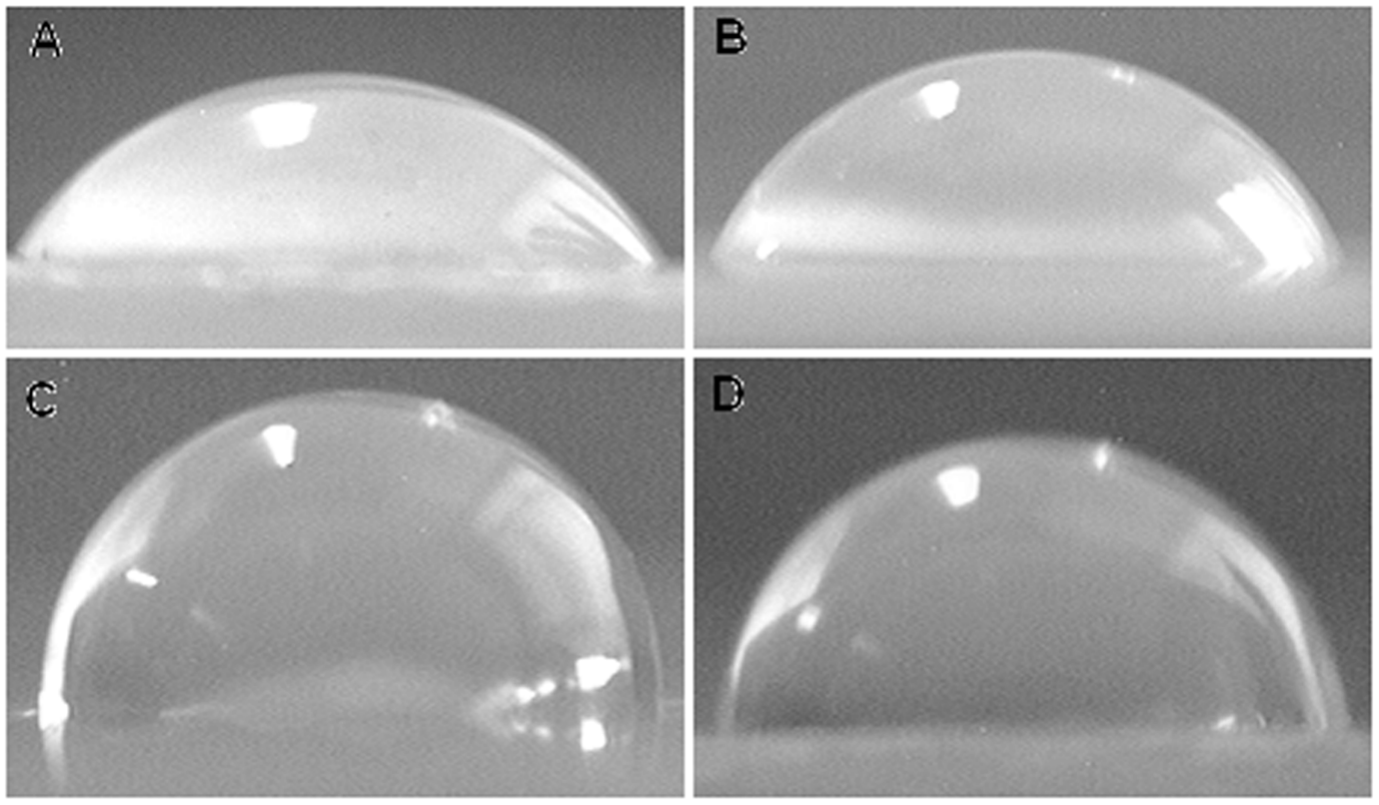
Contact angle images of the synthesized LDHs. A: S-Ni_0.1_MgAl, B: S-Ni_0.1_MgAl-Nd, C: S-Ni_0.1_MgAl-Ce and D: S-Ni_0.1_MgAl-La.

Figures 4 A-D illustrate the TEM images of S-Ni_0.1_MgAl, S-Ni_0.1_MgAl-Nd, S-Ni_0.1_MgAl-Ce and S-Ni_0.1_MgAl-La, respectively. All LDHs show the flake particle morphology. Figures 5 A-D show the contact angle images of S-Ni_0.1_MgAl and RE-doped LDHs, for confirming the effect of different rare earth ions La, Ce and Nd on the hydrophilicity and hydrophobicity of the modified LDHs. The contact angle values of S-Ni_0.1_MgAl, S-Ni_0.1_MgAl-Nd, S-Ni_0.1_MgAl-Ce and S-Ni_0.1_MgAl-La are 63°, 68°, 94° and 87°, respectively. These values can indicate that the addition of rare earth obviously improved the hydrophobicity of LDHs, especially for S-Ni_0.1_MgAl-Ce and S-Ni_0.1_MgAl-La. One potential reason is that S-Ni_0.1_MgAl-La and S-Ni_0.1_MgAl-Ce have relatively better adsorption effects on the stearate than S-Ni_0.1_MgAl and S-Ni_0.1_MgAl-Nd. The other one is that the inherent nature of different rare earth ions has evident effect on the hydrophobicity of LDHs. Compared with the contact angle value of the pristine EVA copolymer equaling 85° [41], the contact angle of S-Ni_0.1_MgAl-Ce is much larger than that of EVA, while the contact angle of S-Ni_0.1_MgAl-La is very close to that of the pristine EVA.

**Figure 6:**
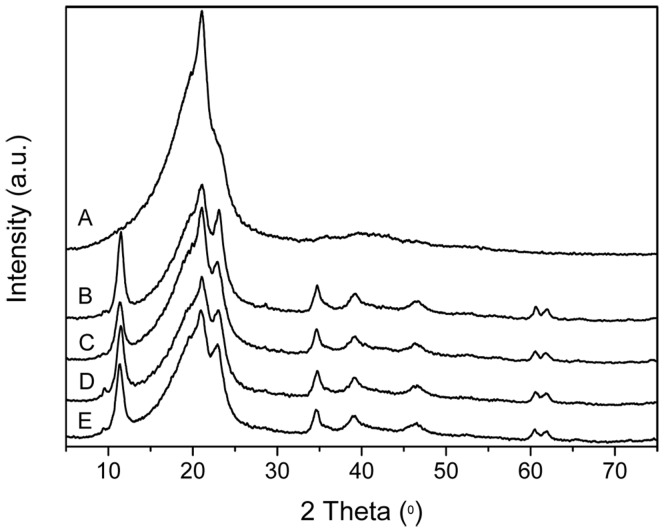
XRD patterns for the pristine EVA and LDHs/EVA composites with a loading of 20 wt% LDHs. A: the pristine EVA, B: S-Ni_0.1_MgAl/EVA, C: S-Ni_0.1_MgAl-Nd/EVA, D: S-Ni_0.1_MgAl-Ce/EVA and E: S-Ni_0.1_MgAl-La/EVA.

### XRD Characterization of Composites

Figures 6 A-E show the XRD patterns of the pristine EVA and LDHs/EVA composites including S-Ni_0.1_MgAl/EVA, S-Ni_0.1_MgAl-Nd/EVA, S-Ni_0.1_MgAl-Ce/EVA and S-Ni_0.1_MgAl-La/EVA with 20 wt% LDHs loading, respectively. All of the above composites show the characteristic reflections of LDHs and the pristine EVA. This result indicates that LDHs are dispersed in EVA matrix.

### Morphological Structures of Composites

**Figure 7:**
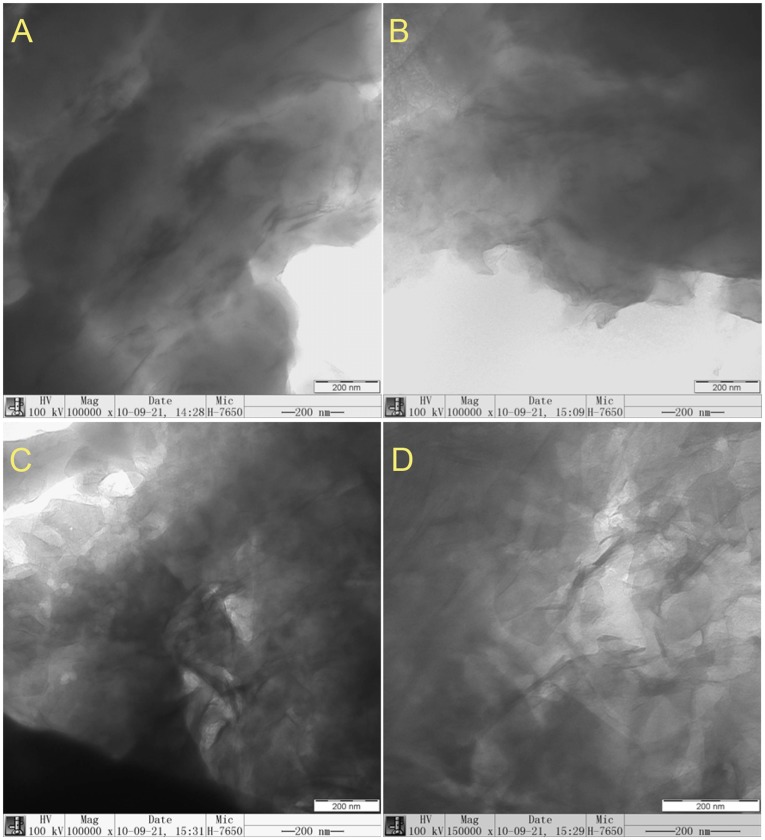
TEM images for LDHs/EVA composites with a loading of 20 wt% LDHs. A: S-Ni_0.1_MgAl/EVA, B: S-Ni_0.1_MgAl-Nd/EVA, C: S-Ni_0.1_MgAl-Ce/EVA and D: S-Ni_0.1_MgAl-La/EVA

**Figure 8:**
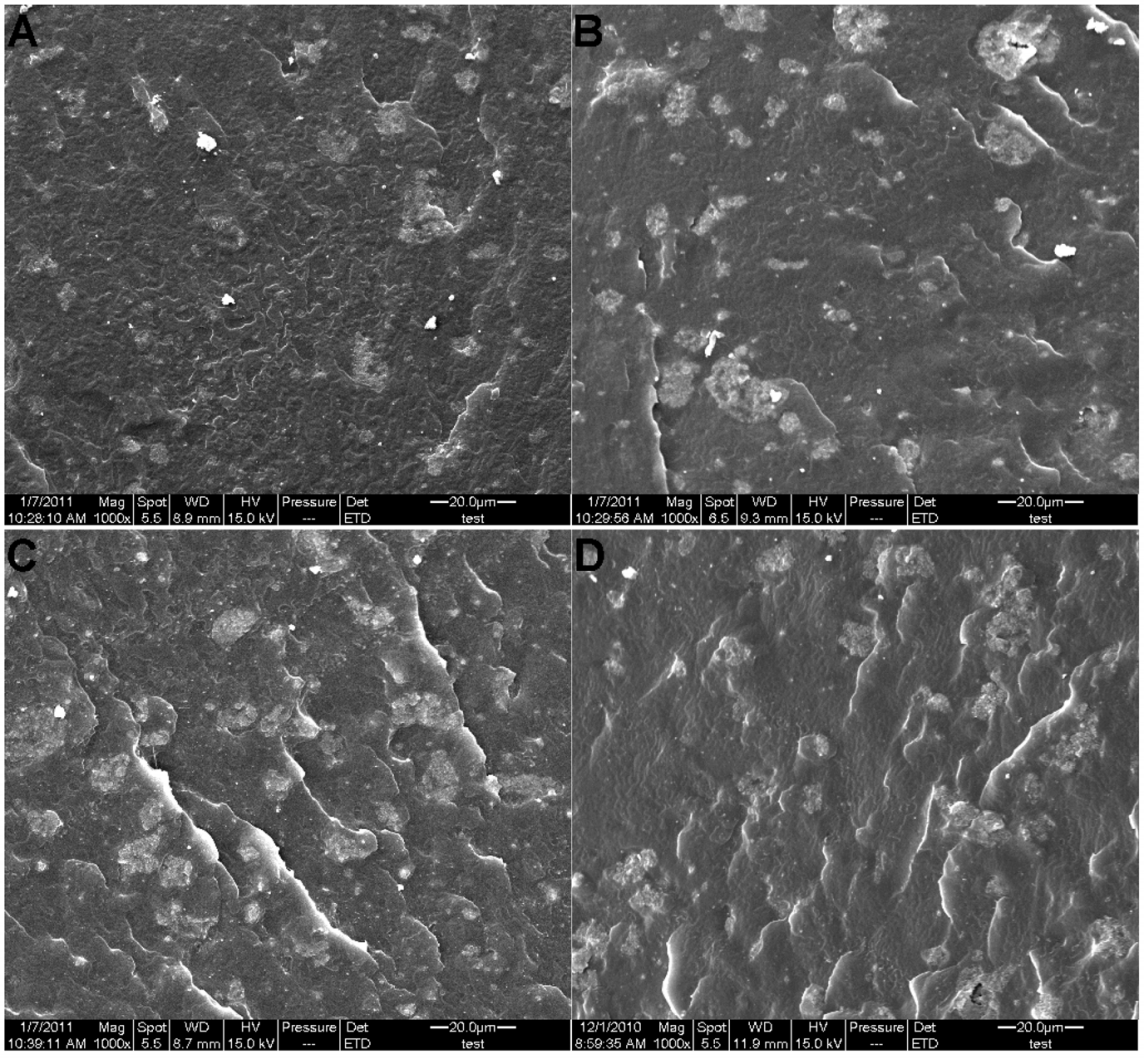
SEM images of the fractured surface for the composites. A: S-Ni_0.1_MgAl-La/EVA, B: S-Ni_0.1_MgAl-Ce/EVA, C: S-Ni_0.1_MgAl-Nd/EVA and D: S-Ni_0.1_MgAl/EVA.

To determine the dispersion of LDHs in EVA copolymer, Figures 7 and 8 show the TEM and SEM images of four LDHs/EVA composites with the 20 wt% loading of LDHs, respectively. It is clear that S-Ni_0.1_MgAl and S-Ni_0.1_MgAl-Nd display more agglomerations than S-Ni_0.1_MgAl-Ce and S-Ni_0.1_MgAl-La in the EVA matrix. While, S-Ni_0.1_MgAl-La shows more homogeneous dispersion in S-Ni_0.1_MgAl-La/EVA (see Figure 7). From Figure 8, it can be seen that the interfaces between EVA and S-Ni_0.1_MgAl-Ce, S-Ni_0.1_MgAl-Nd and S-Ni_0.1_MgAl were defined. Furthermore, the fracture surfaces of composites S-Ni_0.1_MgAl-Nd/EVA and S-Ni_0.1_MgAl/EVA were unsmooth, especially for S-Ni_0.1_MgAl-Nd/EVA. On the contrary, the fracture surface of composite S-Ni_0.1_MgAl-La/EVA was the smoothest. And the interface between EVA and S-Ni_0.1_MgAl-La phase was not clear. Therefore, the SEM micrographs of fractured surface indicate that S-Ni_0.1_MgAl-La shows not only the more uniform dispersion, but also the best interfacial compatibility in the EVA matrix due to the addition of trace rare earth La ions. This result confirms that LDHs/EVA has better interfacial compatibility when the hydrophobicity of LDHs is closer to the pristine EVA, rather than that LDHs with larger hydrophobicity can present better interfacial compatibility. Based on the above fact, S-Ni_0.1_MgAl-La/EVA and S-Ni_0.1_MgAl-Ce/EVA can be expected to show the better flame retardancy and mechanical properties, especially for S-Ni_0.1_MgAl-La/EVA.

### Combustion Behavior of Composites

**Figure 9:**
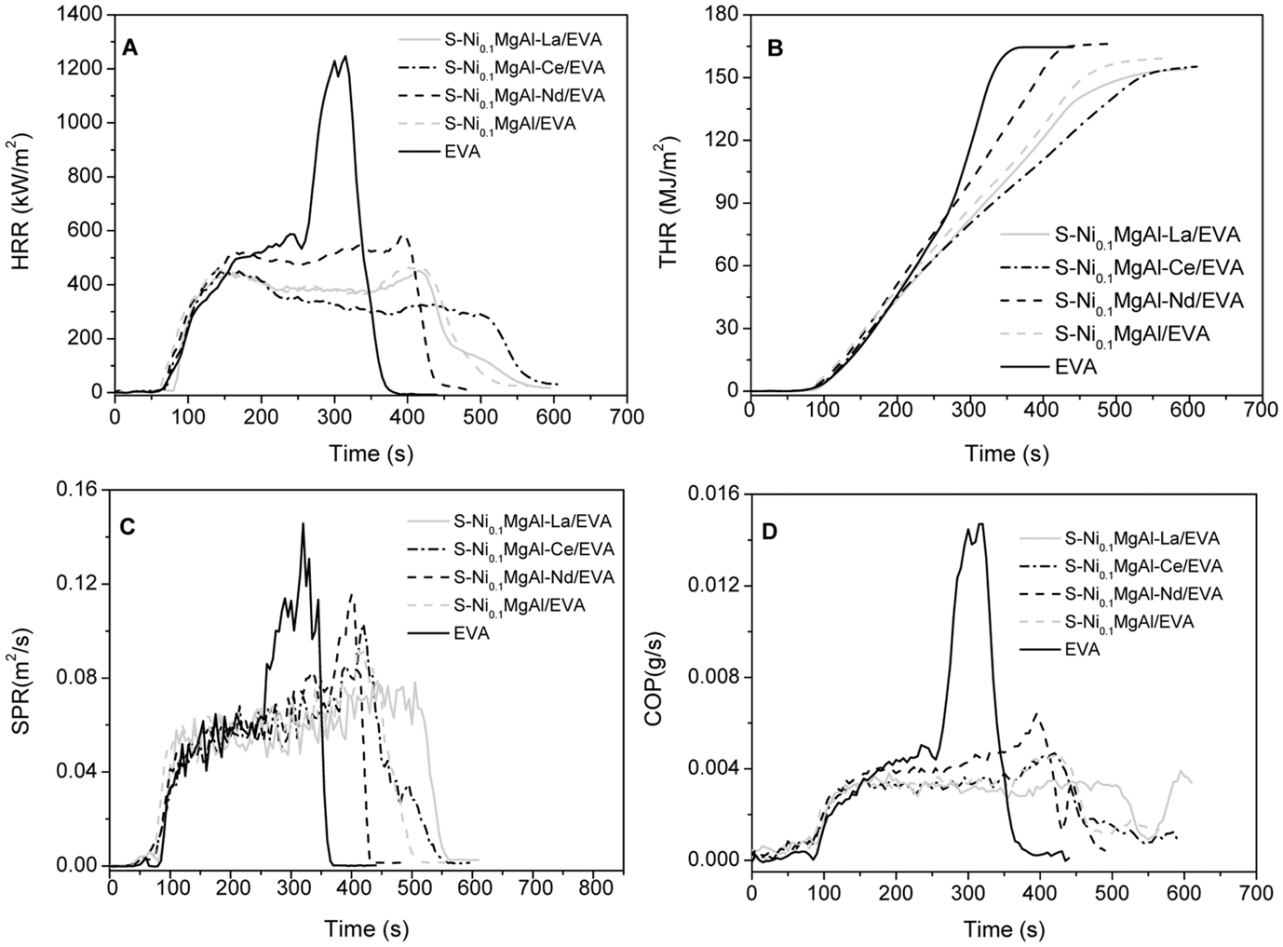
Cone calorimetric results of the pristine EVA and its composites with 20 wt.% loading of LDHs. A: Heat release rate (HRR) curves, B: Total heat release (THR) curves, C: Smoke production rate (SPR) curves and D: Production of CO (COP) curves.

The fire and smoke properties of composites with 20 wt.% loading of LDHs were evaluated using the cone calorimeter. Figure 9 A shows the heat release rate (HRR) for the EVA and its composites. It can be seen that the peak value of HRR (pk-HRR) for the pristine EVA is 1247 kW/m^2^, while the pk-HRR for S-Ni_0.1_MgAl-La/EVA, S-Ni_0.1_MgAl-Ce/EVA, S-Ni_0.1_MgAl-Nd/EVA and S-Ni_0.1_MgAl/EVA composites are 450, 452, 591 and 464 kW/m^2^, respectively. This result indicates that the addition of the four LDHs evidently decreases the HRR of composites. Compared with the ignition times (T_ign_) of the pristine EVA which is 62 s, it is worth noticing that T_ign_ of S-Ni_0.1_MgAl-La/EVA, S-Ni_0.1_MgAl-Ce/EVA and S-Ni_0.1_MgAl-Nd/EVA were postponed to 83 s, 70 s and 67 s, respectively, whereas that of S-Ni_0.1_MgAl/EVA was shorten to 59 s. Therefore, S-Ni_0.1_MgAl-La/EVA is the better flame retardancy composite because the harder burning materials need longer ignition times. Furthermore, the fire performance index (FPI) as another important fire resistance parameter is defined as the ratio of pk-HRR to T_ign_. FPI values of S-Ni_0.1_MgAl-La/EVA, S-Ni_0.1_MgAl-Ce/EVA, S-Ni_0.1_MgAl-Nd/EVA and S-Ni_0.1_MgAl/EVA are 5, 6, 9 and 9 kW/m^2^s, respectively. FPI values of all of the above composites are obviously smaller than that of the pristine EVA (20 kW/m^2^s). Therefore, FPI values also confirm that S-Ni_0.1_MgAl-La/EVA has the highest flame retardant efficiency. In other word, the lower FPI values mean the better fire safe materials [24]. In addition, as can be seen from the total heat release (THR) curves in Figure 9 B, the THR values of composites are smaller than that of the pristine EVA except S-Ni_0.1_MgAl-Nd/EVA. The above all typical parameters obtained from the cone calorimeter experiment indicate that S-Ni_0.1_MgAl-La has the best flame retardant efficie ncy, while S-Ni_0.1_MgAl-Nd shows the least flame retardancy.

**Figure 10:**
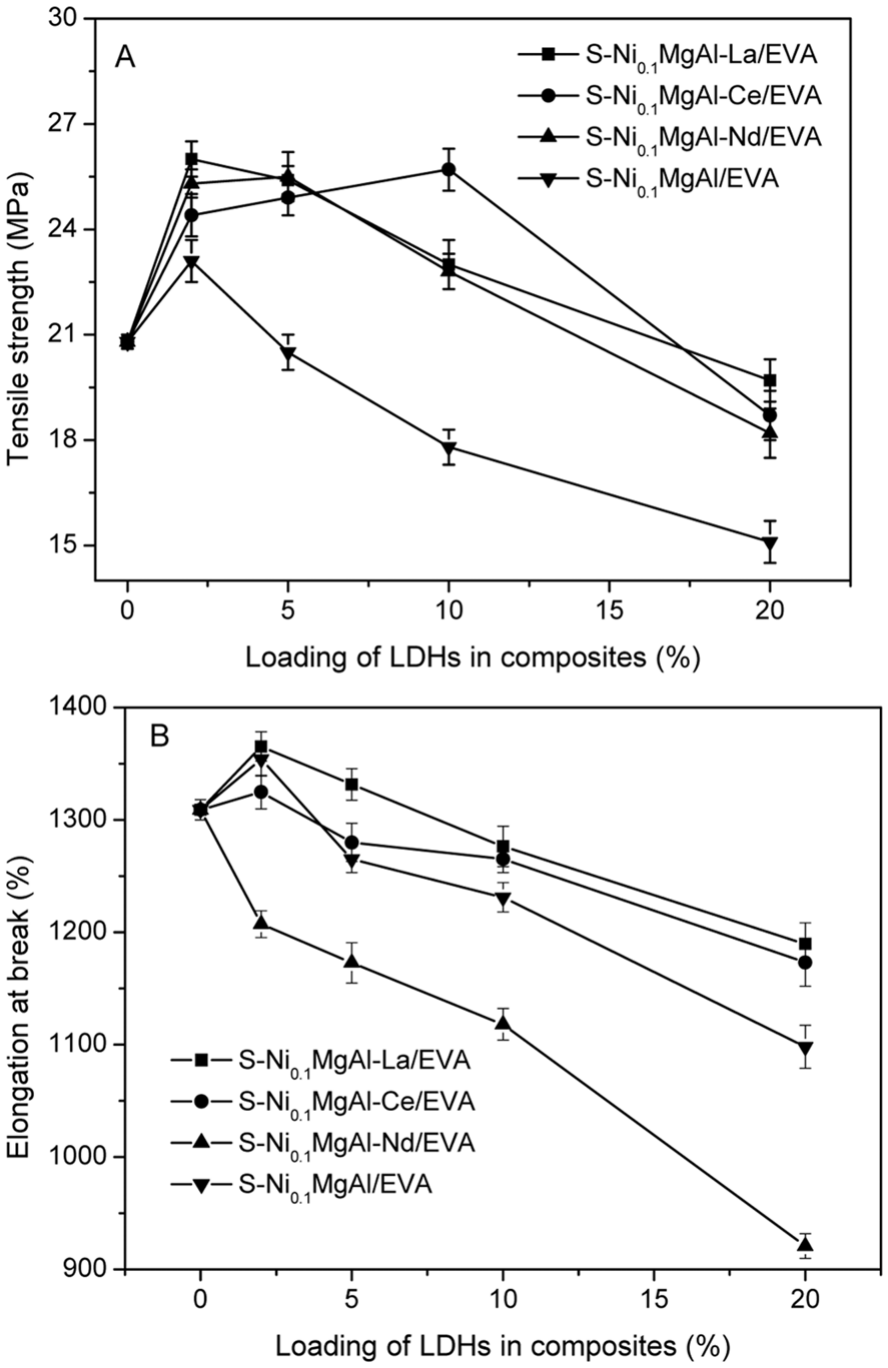
Tensile strength and the elongation at break of composites with different contents of LDHs. A: Tensile strength curves and B: Elongation at break curves.

The release of smoke and toxic gas is considered as another important parameter for the flame retarded materials. Figures 9 C and D show the smoke production rate (SPR) and the production rate of CO (COP) curves of the pristine EVA and its composites with 20 wt.% loading of LDHs, respectively. The peak values of SPR (pk-SPR) for EVA, S-Ni_0.1_MgAl-La/EVA, S-Ni_0.1_MgAl-Ce/EVA, S-Ni_0.1_MgAl-Nd/EVA and S-Ni_0.1_MgAl/EVA are 0.146, 0.077, 0.102, 0.114 and 0.092 m^2^/s, respectively. At the same time, the peak values of COP (pk-COP) for EVA, S-Ni_0.1_MgAl-La/EVA, S-Ni_0.1_MgAl-Ce/EVA, S-Ni_0.1_MgAl-Nd/EVA and S-Ni_0.1_MgAl/EVA are 0.0147, 0.0037, 0.0046, 0.0064 and 0.0047 g/s, respectively. The values of SPR and COP for all composites were significantly reduced compared with the pristine EVA, especially for S-Ni_0.1_MgAl-La/EVA. This result is similar to those of HRR and THR. The above cone calorimeter data provide evidence that S-Ni_0.1_MgAl-La/EVA performs the best both flame retardancy and smoke/toxic gas suppression.

### Mechanical Properties of Composites

Figures 10 A and B show the tensile strength and the elongation at break for the pristine EVA and its composites with 2 wt.%, 5 wt.%, 10 wt.% and 20 wt.% loading of LDHs, respectively. As shown in Figure 10 A, when 2 wt. % content of LDHs was added to the EVA matrix, the tensile strength values of all composites show a significant increase compared with that of the pristine EVA (20.8 MPa). In particular, the tensile strength of S-Ni_0.1_MgAl-La/EVA was increased to the maximum value of 26.0 MPa. Furthermore, the tensile strength values of S-Ni_0.1_MgAl-La/EVA, S-Ni_0.1_MgAl-Ce/EVA and S-Ni_0.1_MgAl-Nd/EVA are more than 4 MPa in comparison with those of the pristine EVA and S-Ni_0.1_MgAl/EVA when the addition of 5 wt.% RE-doped LDHs to EVA. The tensile strength values of S-Ni_0.1_MgAl-La/EVA, S-Ni_0.1_MgAl-Ce/EVA and S-Ni_0.1_MgAl-Nd/EVA are 5 MPa more than that of S-Ni_0.1_MgAl/EVA when the loading of LDHs was kept in 10 wt% of composites. Further, the tensile strength value of S-Ni_0.1_MgAl-Ce/EVA was increased to 25.7 MPa which is 7.9 MPa greater than that of 17.8 MPa of S-Ni_0.1_MgAl/EVA when 10 wt. % content of S-Ni_0.1_MgAl-Ce was added to the EVA matrix. In addition, it is worth noticing that when the addition of 20 wt.% LDHs is chosen as a maximum content point of comparison, the tensile strength value of S-Ni_0.1_MgAl-La/EVA at 19.7 MPa is only slightly lower than that of the pristine EVA. However, the tensile strength of S-Ni_0.1_MgAl/EVA with 20 wt.% loading of S-Ni_0.1_MgAl decreases to the minimum value (15.1 MPa). Therefore, our conclusion is that the addition of rare earth ions La, Ce and Nd significantly improve the strength of composites in comparison with S-Ni_0.1_MgAl/EVA, especially for the addition of La ions.

As can be seen from Figure 10 B, the addition of the rare earth La, Ce and Nd obviously affects the elongation at break of composites. For instance, except for S-Ni_0.1_MgAl-Nd/EVA, the elongation at break values of other composites show an increase compared with that of the pristine EVA equaling 1309% when 2 wt. % content of LDHs is added to the EVA matrix. In addition, the elongation at break of all composites was decreased as the increase of LDHs content when the loading of LDHs was kept from 5 to 20 wt.% of composite. As expected, the composite S-Ni_0.1_MgAl-La/EVA presents the greatest the elongation at break values among all composites with different contents of LDHs. This result indicates that S-Ni_0.1_MgAl-La/EVA has the best ductility due to the good interface compatibility between S-Ni_0.1_MgAl-La and the EVA matrix. On the contrary, S-Ni_0.1_MgAl-Nd/EVA has the lowest ductility. It is agreement with the result of the worst interface compatibility between S-Ni_0.1_MgAl-Nd and the EVA matrix.

### Summary

XRD results indicate that the addition of rare earth ions not only reduces the crystallinity of the LDHs, but also the basal spacing of RE-doped LDHs is expanded in comparison with S-Ni_0.1_MgAl. IR results confirm that S-Ni_0.1_MgAl-La has better adsorption effect on stearate due to the addition of La ions. Contact angle values show that the addition of rare earth obviously improves the hydrophobicity of LDHs, especially for S-Ni_0.1_MgAl-Ce and S-Ni_0.1_MgAl-La. Furthermore, LDHs/EVA has better interfacial compatibility when the hydrophobicity of LDHs is closer to the hydrophobicity of the pristine EVA. The TEM and SEM images of composites indicate that S-Ni_0.1_MgAl-La has the best interfacial compatibility in the EVA matrix. At the same time, cone calorimeter data and mechanics test results provide the evidence that S-Ni_0.1_MgAl-La/EVA performs the best flame retardancy and mechanical properties.

## Materials and Methods

### Preparation of the Surface Modified Ni-containing LDHs and RE-doped Ni-containing LDHs

All chemicals used in the preparation were analytical grade without further purification. EVA (VA-28%) was purchased from Samsung Co. in Korea. Deionized water was made by Milli-Q academic water purification system in our Lab.

The synthesis of stearate sodium surface modified Ni-containing LDHs was implemented by a coprecipitation method coupled with the microwave hydrothermal treatment. In a four-necked flask (l L), a mixed aqueous solution containing 6 ml 1 M Ni(NO_3_)_2_, 174 ml 1 M Mg(NO_3_)_2_ and 60 ml 1 M Al(NO_3_)_3_ with a Ni^2+^:Mg^2+^:Al^3+^ molar ratio of 0.1∶2.9∶1 was dropwise added to 100 ml of deionized water at 70°C under continuous magnetic stirring, while the pH was adjusted to the range of 8–9 by adding NaOH-Na_2_CO_3_ mixed solution (0.6 M NaOH and 0.45 M Na_2_CO_3_). After the titration, a heavy suspension gel was obtained and transfered to the beaker (l L). Then, the beaker with suspension gel was put in a microwave oven (XH-300A, the maximum power of 1000 W and a frequency of 2.45 GHz) and crystallized at 70°C for 30 min. The temperature was controlled by a temperature feedback monitoring system with dual IR sensors. The obtained precipitate was washed by the deionized water to pH 7, and then filtered. The synthesized sample was dried under air atmosphere at 70°C.

Preparation of the modified RE-doped Ni-containing LDHs. In a four-necked flask (l L), a mixed aqueous solution containing 6 ml 1 M Ni(NO_3_)_2_, 174 ml 1 M Mg(NO_3_)_2_, 30 ml 0.1 M RE(NO_3_)_3_ (RE = La, Ce, Nd) and 57 ml 1 M Al(NO_3_)_3_ with a Ni^2+^:Mg^2+^:RE^3+^:Al^3+^ molar ratio of 0.1∶2.9∶0.05∶0.95 was dropwise added to 100 ml of deionized water at 70°C under continuous magnetic stirring, while the pH was adjusted to the range of 8–9 by adding NaOH-Na_2_CO_3_ mixed solution (0.6 M NaOH and 0.45 M Na_2_CO_3_). All the following synthetic processes are in accordance with the synthesis of stearate sodium surface modified Ni-containing LDHs.

0.15 g stearate sodium and the above synthesized LDHs 10 g were dispersed into 200 ml deionized water. The mixture was vigorously stirred at 70°C with the microwave irradiation for 30 min. Then the precipitate was washed to pH 7 with the 70°C hot water to eliminate the excessive stearate sodium, and then filtered. The surface modified LDHs were dried in an oven for 4 h at 70°C. The resulting surface modified Ni-containing LDHs and RE-doped Ni-containing LDHs were designated as S-Ni_0.1_MgAl, S-Ni_0.1_MgAl-La, S-Ni_0.1_MgAl-Ce and S-Ni_0.1_MgAl-Nd, respectively.

### Preparation of LDHs/EVA Composites

The LDHs/EVA composites were prepared via melt blending at 150°C in an RM-200A torque rheometer for 10 min with a rotor speed of 60 rpm. In order to obtain the optimum performance of composites, several formulations with the additive amount of LDHs from small to large rations (2 wt.%, 5 wt.%, 10 wt.% and 20 wt.% mass fraction of LDHs content) were systematically investigated. The composites are named as S-Ni_0.1_MgAl/EVA, S-Ni_0.1_MgAl-La/EVA, S-Ni_0.1_MgAl-Ce/EVA and S-Ni_0.1_MgAl-Nd/EVA, respectively.

### Characterization Techniques

Thermal analysis were carried out with a thermogravimetric analyzer (TGA pyris 1 from Perkin Elmer) using a constant heating rate of 10°C/min under nitrogen atmosphere from 50 to 800°C. X-ray diffraction (XRD) experiments were performed by using a D/MAX 2200 diffractometer with *λ*  = 1.5406 Å for angle 2*θ  = *5–75°. Data were collected at the rate of 4 °/min and step of 0.02 ° with Cu Kα irradiation operated at 40 kV and 45 mA. Infrared (IR) spectra of samples were collected using Nicolet FTIR360 spectrometer (KBr pellet method, 4 cm^−1^ resolution). Contact angles (CA) were measured using JC2000A contact angle measurer. All LDHs were pressed into disks with a diameter of 11 mm and a thickness of 0.4 mm. Each disk was dripped by 3 µL of deionized water. The observation of all water drops maintains for 30 sec. The measurement for each sample repeats five times. For transmission electron microscope (TEM) observation, the specimens were examined in a HITACHI 1H-7650 transmission electron microscope operated at an accelerating voltage of 100 kV. Scanning electron microscope (SEM) observations were carried out on an FEI-Sirion with a field emission at 20 kV. The combustion behaviors of all samples were tested by using a Stanton Redcroft cone calorimeter at an incident heat flux of 50 kWm^−2^ according to ISO 5660-1 standard. Determination of elongation at break and tensile strength of composites were performed by a RGD-20A material test machine (produced by Shenzhen Regear Instrument Cooperation, China), according to the national standard GB/T 16421-1996.
